# Immune Microenvironmental Analysis of Intraductal Papillary Neoplasms of the Bile Duct in Occupational Cholangiocarcinoma: Associations with Tumor Size

**DOI:** 10.3390/cancers18162539

**Published:** 2026-08-07

**Authors:** Masahiko Kinoshita, Shoji Kubo, Kimika Kato, Daisuke Inoue, Takuto Yasuda, Kosuke Hatta, Atsushi Sugimoto, Ryota Tanaka, Jun Tauchi, Sadaaki Nishimura, Genki Watanabe, Kohei Nishio, Hiroji Shinkawa, Takeaki Ishizawa, Yasunori Sato

**Affiliations:** 1 Department of Hepato-Biliary-Pancreatic Surgery, Osaka Metropolitan University Graduate School of Medicine, Osaka 545-8585, Japanjnt_ch@hotmail.co.jp (J.T.);; 2 Health Education Course, Department of Education, Faculty of Education, Shitennoji University, 3-2-1 Gakuenmae, Habikino 583-8501, Japan; 3 Department of Human Pathology, Kanazawa University Graduate School of Medical Sciences, Kanazawa 920-8640, Japan

**Keywords:** intraductal papillary neoplasm of the bile duct, occupational cholangiocarcinoma, tumor immune microenvironment, immune checkpoint inhibitor

## Abstract

**Background/Objectives:** Intraductal papillary neoplasm of the bile duct (IPNB) is a premalignant lesion of cholangiocarcinoma. Currently, its tumor immune microenvironment (TIME) remains poorly characterized. Occupational cholangiocarcinoma provides a unique setting wherein multiple IPNBs arise in a background of chronic biliary injury caused by organic solvents. This study evaluated TIME alternations in IPNB lesions in occupational cholangiocarcinoma, focusing on histological subclassifications, phenotypes, invasive status, and tumor size. **Methods:** Thirteen IPNB lesions in six patients with occupational cholangiocarcinoma were immunohistochemically stained for CD8, CD163, programmed death protein 1 (PD-1), and programmed death ligand 1 (PD-L1). CD8-, CD163-, and PD-1-positive cells were counted in the most densely infiltrated areas. PD-L1 expression was assessed using the combined positive score (CPS), and associations with each status were analyzed. **Results:** Within the occupational IPNB cohort, type 2 IPNBs demonstrated significantly higher CD8, CD163, and PD-1 expression levels than type 1 IPNBs. Invasive lesions showed significantly higher CD8 expression and CPS than noninvasive lesions. Tumor size was positively correlated with CD163 expression (R = 0.504), CD8 expression (R = 0.627), and CPS (R = 0.569) in the TIME. When stratified by tumor size (<10 vs. ≥10 mm), lesions ≥ 10 mm in size demonstrated significantly higher CD163 expression and CPS. Notably, all lesions with CPS ≥1 were ≥10 mm in size. **Conclusions:** In IPNB arising from occupational cholangiocarcinoma, tumor enlargement is associated with progressive TIME alterations. Lesions ≥ 10 mm in size may represent a threshold at which immunoediting and invasive potential become more prominent.

## Simple Summary

In this research we addressed the tumor immune microenvironment (TIME) of intraductal papillary neoplasm of the bile duct (IPNB) in occupational cholangiocarcinoma characterized by chronic biliary injury due to organic solvent exposure. The TIME in IPNB remains poorly characterized, particularly regarding associations with histological subclassification, phenotype, invasive status, and tumor size. We analyzed 13 IPNB lesions from six occupational cholangiocarcinoma patients using immunohistochemical staining for CD8, CD163, programmed death protein 1 (PD-1), and programmed death ligand 1 (PD-L1), with PDL1 expression evaluated by the combined positive score (CPS). Positive immune cells were quantified in the most infiltrated areas. Type 2 IPNBs showed higher CD8, CD163, and PD-1 expression levels than type 1 lesions, whereas invasive lesions exhibited increased CD8 expression and CPS compared with noninvasive lesions. Tumor size correlated positively with immune markers, and lesions ≥ 10 mm demonstrated significantly more immunosuppressive features, suggesting progressive immune remodeling and a potential threshold for malignant progression.

## 1. Introduction

Intraductal papillary neoplasm of the bile duct (IPNB) is a premalignant lesion of cholangiocarcinoma characterized by papillary epithelial proliferation within the bile duct lumen [1]. It is categorized into distinct subclassifications (i.e., types 1 and 2) [2,3,4] and phenotypes (e.g., pancreatobiliary, intestinal, gastric, and oncocytic types) [1,5,6], reflecting differences in morphology, mucin expression, and biological behavior. In the carcinogenic pathway of cholangiocarcinoma, carcinogens induce micro-IPNBs (≤5 mm), which then develop into ordinary IPNBs and subsequently progress into invasive IPNBs [7]. Although the clinicopathological features and histological progression of IPNB have been increasingly characterized [8,9], its tumor immune microenvironment (TIME) remains poorly understood. In particular, little is known about how immune-related molecules (e.g., CD8, CD163, programmed death protein 1 [PD-1], and programmed death ligand 1 [PD-L1]) are modulated during tumor growth and progression and whether immune escape mechanisms emerge at specific stages of IPNB development.

Occupational cholangiocarcinoma represents a unique disease entity characterized by chronic bile duct injury and widespread epithelial alterations in the large bile ducts, presumably caused by exposure to organic solvents, including 1,2-dichrolopropane (DCP) and dichrolomethane (DCM) [8]. In patients with occupational cholangiocarcinoma, multicentric biliary cancerous and/or precancerous lesions, including multiple biliary intraepithelial neoplasia and IPNBs, are frequently observed, reflecting a field cancerization phenomenon and a multistep carcinogenic process [10,11,12,13]. Occupational cholangiocarcinoma exhibits an exceptionally high tumor mutational burden (TMB) [12]. In previous studies on occupational cholangiocarcinoma, all invasive carcinoma lesions exhibited frequent expression of immune exhaustion molecules, including PD-1 and PD-L1, with half of IPNB lesions presenting their expression [14,15]. Based on these backgrounds, the favorable impact of immune checkpoint inhibitors on unresectable recurrent occupational cholangiocarcinoma has been reported [16]. However, it remains unclear at which stage of biliary neoplastic progression these immunological alterations arise.

Therefore, the present study analyzed the TIME of IPNB lesions arising in patients with occupational cholangiocarcinoma, focusing on histological subclassification, phenotype, invasive status, and tumor size. This study aimed to clarify the emergence of immune escape mechanisms during IPNB progression and to explore the malignant potential associated with tumor growth.

## 2. Materials and Methods

### 2.1. Study Cohort

The study population comprised 22 patients who developed cholangiocarcinoma after long-term, high-concentration occupational exposure to DCP and/or DCM at a printing company in Osaka, Japan. Out of the 22 patients, 21 were officially recognized as having occupational cholangiocarcinoma by the Ministry of Health, Labour and Welfare [12]. One patient (patient 5) was not officially certified because he did not apply for workers’ compensation due to his own wishes and the absence of a legally authorized representative. However, this patient was considered to have occupational cholangiocarcinoma because of his history of high-concentration exposure to DCP and DCM while working at the printing company for 6 years and 1 month, together with the typical clinicopathological features of occupational cholangiocarcinoma [13]. Among the 22 patients, seven had pathologically identifiable IPNB lesions in the large bile ducts identified in surgical or autopsy specimens. One patient was excluded because the amount of tissue available for immunohistochemical evaluation was inadequate. Consequently, 13 IPNB lesions from these six patients were included in the present study. IPNB was pathologically diagnosed using surgical or autopsy specimens based on the characteristic findings of intraductal papillary or villous biliary neoplasms covering delicate fibrovascular stalks, with or without invasiveness. In accordance with the World Health Organization classification, only intrahepatic lesions were included in this study because the definition of extrahepatic IPNB remains controversial and may overlap with papillary-type extrahepatic cholangiocarcinoma [1].

Histological subclassifications of IPNB were classified pathologically. The first type (type 1 IPNB) is histologically similar to intraductal papillary neoplasms of the pancreas and typically develops in the intrahepatic bile ducts, whereas the second type (type 2 IPNB) has a more complex histological architecture with irregular papillary branching bile ducts and is frequently associated with invasive carcinoma [2,3,4,8].

Meanwhile, epithelial cellular phenotypes were further classified into pancreatobiliary, gastric, intestinal, and oncocytic types according to the most prevalent morphological pattern [1,5].

Among the 13 IPNB lesions, immunohistochemical analyses were performed to determine TIME characteristics. Moreover, the characteristics that were analyzed were compared based on histological subclassifications (type 1 or 2), epithelial cellular phenotypes (pancreatobiliary, gastric, intestinal, or oncocytic type), presence of invasiveness (invasive or noninvasive IPNB), and tumor diameter.

This study was approved by the Ethics Committee of Osaka Metropolitan University (no. 2022-116) and performed following the Declaration of Helsinki. All patients provided written informed consent.

### 2.2. TIME Analyses

Immunohistochemical analyses were performed using antibodies against CD8 (clone C8/144B, 1:20; Dako, Tokyo, Japan), CD163 (clone EPR19518, 1:500; Abcam, Tokyo, Japan), PD-1 (clone NAT105, 1:100; Abcam), and PD-L1 (clone 28-8, 1:500; Abcam) to evaluate the TIME. After deparaffinization, antigen retrieval was performed by autoclaving in Universal HIER Antigen Retrieval Reagent (Abcam, Tokyo, Japan) for PD-L1 and by microwaving in Tris-EDTA (pH 9.0) for CD8, CD163 and PD-1. After blocking endogenous peroxidase, sections were incubated with primary antibodies for 1 h at room temperature. Subsequently, they were incubated with the Rabbit-specific IHC Polymer Detection Kit HRP/DAB (Abcam) for PD-L1 staining and Histofine Simple Stain MAX PO (Nichirei, Tokyo, Japan) for CD8, CD163, and PD-1 staining. Color development was performed using 3,3′-diaminobenzidine tetrahydrochloride, followed by counterstaining with hematoxylin. Negative controls were prepared by replacing the primary antibody with nonimmune serum.

Immunohistochemical evaluation was independently performed by three investigators (Y.S., M.K., and S.K.) in a non-blinded manner. For CD8, CD163, and PD-1, the hotspot showing the highest density of positive cells within the tumor was selected, and positive cells were counted in one high-power field (0.238 mm^2^). Only intratumoral areas were evaluated. Tumor margins and lymphoid follicles occasionally present within the tumor stroma were excluded from the analysis because they could artificially increase immune cell counts.

PD-L1 expression was evaluated using the combined positive score (CPS), which was calculated as previously described [17]. Briefly, the CPS was defined as the number of PD-L1-positive cells (including tumor cells, lymphocytes, and macrophages) divided by the total number of viable tumor cells ×100. In contrast to CD8, CD163, and PD-1 evaluation, CPS assessment included immune cells located within and immediately adjacent to the tumor.

### 2.3. Statistical Analysis

Continuous variables were analyzed using Student’s *t*-test or the Mann–Whitney U test, as appropriate. Associations in 2 × 2 contingency tables were evaluated using Fisher’s exact test. Correlations between tumor size and immune markers were assessed using Pearson’s correlation coefficient. Statistical significance was considered at *p* < 0.05.

## 3. Results

### 3.1. Background Characteristics and Pathological Finding of 13 IPNBs in 6 Patients

The clinicopathological characteristics of the six patients and 13 IPNB lesions are summarized in Table 1. The median age of the patients was 39 years (range, 31–47 years). Four patients had a history of occupational exposure to DCP alone, whereas two patients had been exposed to DCP and DCM. Five patients underwent surgical resection, whereas one received chemotherapy. In five patients, the primary tumor was located in the intrahepatic bile duct, presenting as invasive IPNB or mass-forming intrahepatic cholangiocarcinoma. The median tumor diameter was 5 mm (range, 1.5–16 mm). Seven lesions were classified as type 1, and six were classified as type 2. Regarding epithelial phenotype, seven lesions were classified as the pancreatobiliary type, four were classified as the gastric type, and two were classified as the oncocytic type. Three lesions contained invasive components (Table 1).

**Figure 1:**
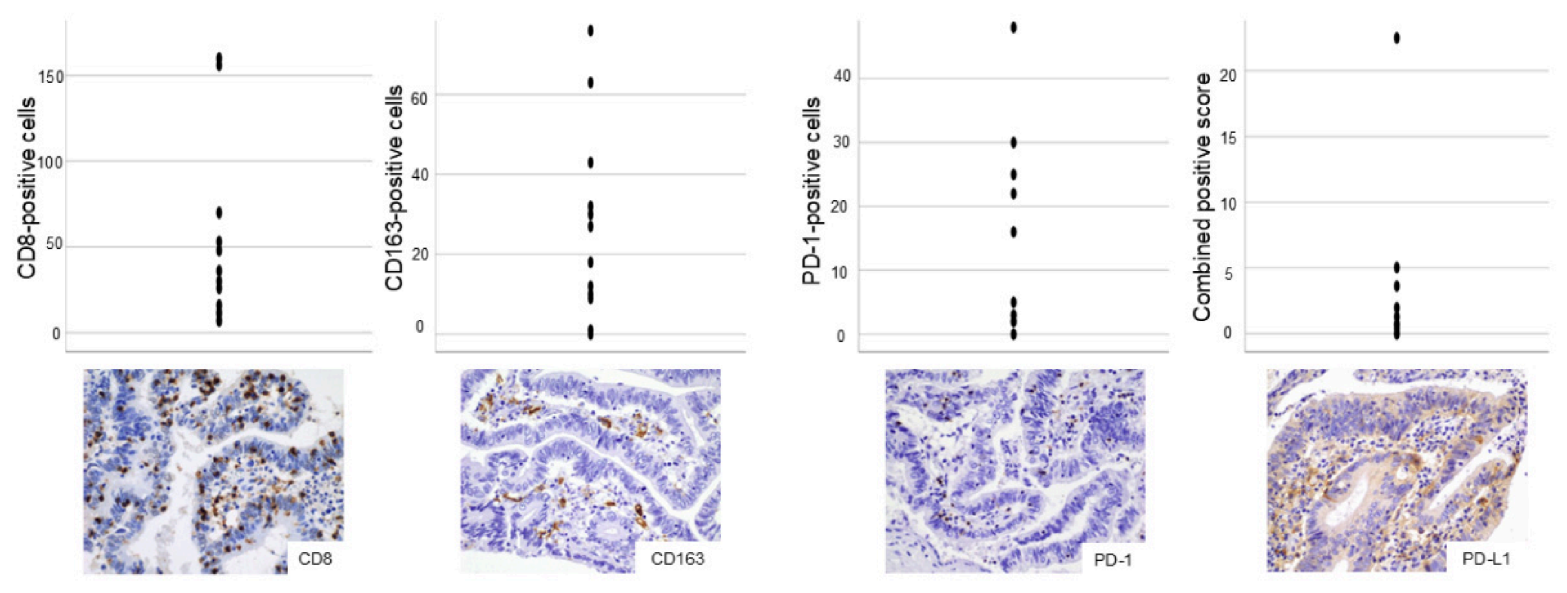
Distribution of immune-related markers in 13 IPNBs. The box plots show the distributions of CD8-, CD163-, and PD-1-positive cells and the PD-L1 CPS in the tumor immune microenvironment of 13 occupational IPNB lesions. Abbreviations: IPNB, intraductal papillary neoplasm of the bile duct; PD-1, programmed cell death protein 1; PD-L1, programmed death ligand 1; CPS, combined positive score.

The median number of CD8-, CD163-, and PD-1-positive cells was 30, 18, and 3, respectively, and the median CPS was 0.70 (Figure 1).

### 3.2. Comparison of the TIME Between Histological Subclassifications

**Figure 2:**
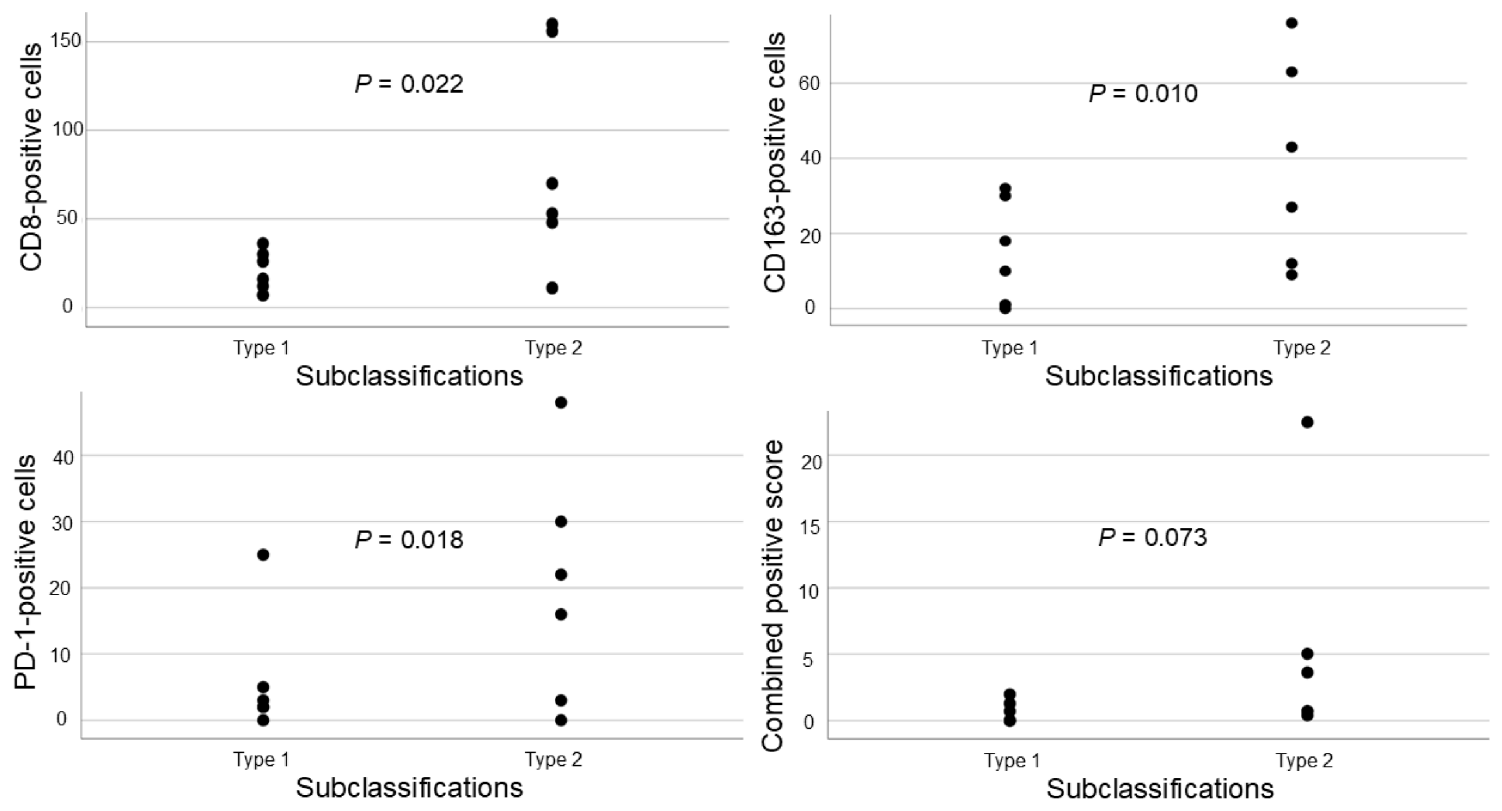
Comparison of the tumor immune microenvironment between type 1 and type 2 IPNBs. Type 2 IPNBs demonstrated significantly higher CD8-, CD163, and PD-1-positive cell infiltration compared with type 1 IPNBs. However, no significant difference was observed in CPS.

Type 2 IPNBs demonstrated significantly higher CD8, CD163 and PD-1 expression in the TIME than type 1 IPNBs (*p* = 0.022, 0.010 and 0.018, respectively). No significant differences were observed in the expression of other immune-related molecules between type 1 and 2 IPNBs (Figure 2).

### 3.3. Comparison of the TIME According to Epithelial Cellular Phenotypes

**Figure 3:**
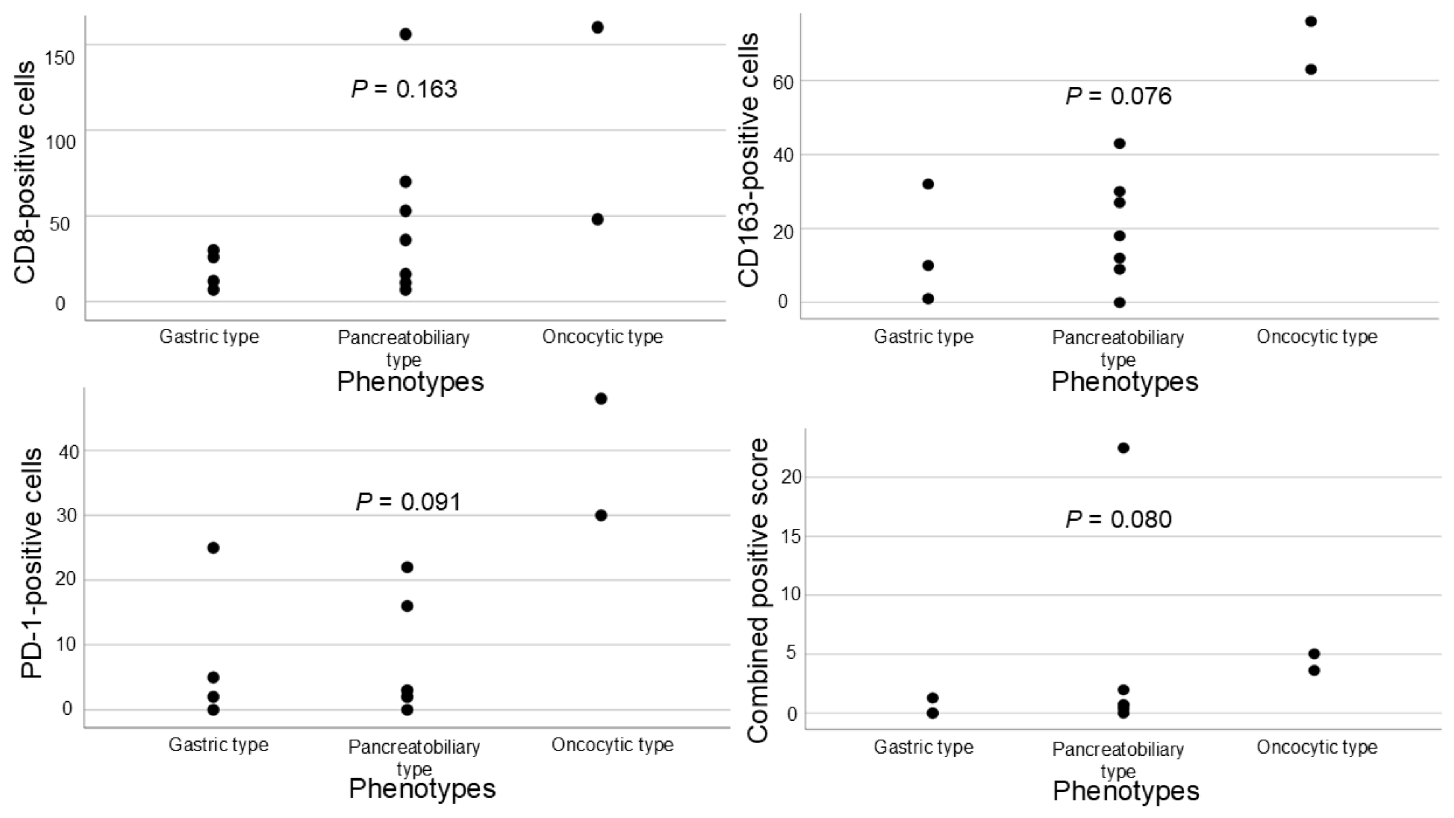
Comparison of the tumor immune microenvironment according to epithelial cellular phenotypes of IPNBs. CD8-, CD163-, and PD-1-positive cells and PD-L1 CPS were compared among gastric, pancreatobiliary, and oncocytic phenotypes. No significant differences were observed among the three groups.

Figure 3 shows a comparison of the TIME according to epithelial cellular phenotypes. As can be seen, no significant differences were observed in the CD8, CD163, or PD-1 expression level or CPS among the gastric, pancreatobiliary, and oncocytic types.

### 3.4. Comparison of the TIME Between Invasive and Noninvasive IPNBs

**Figure 4:**
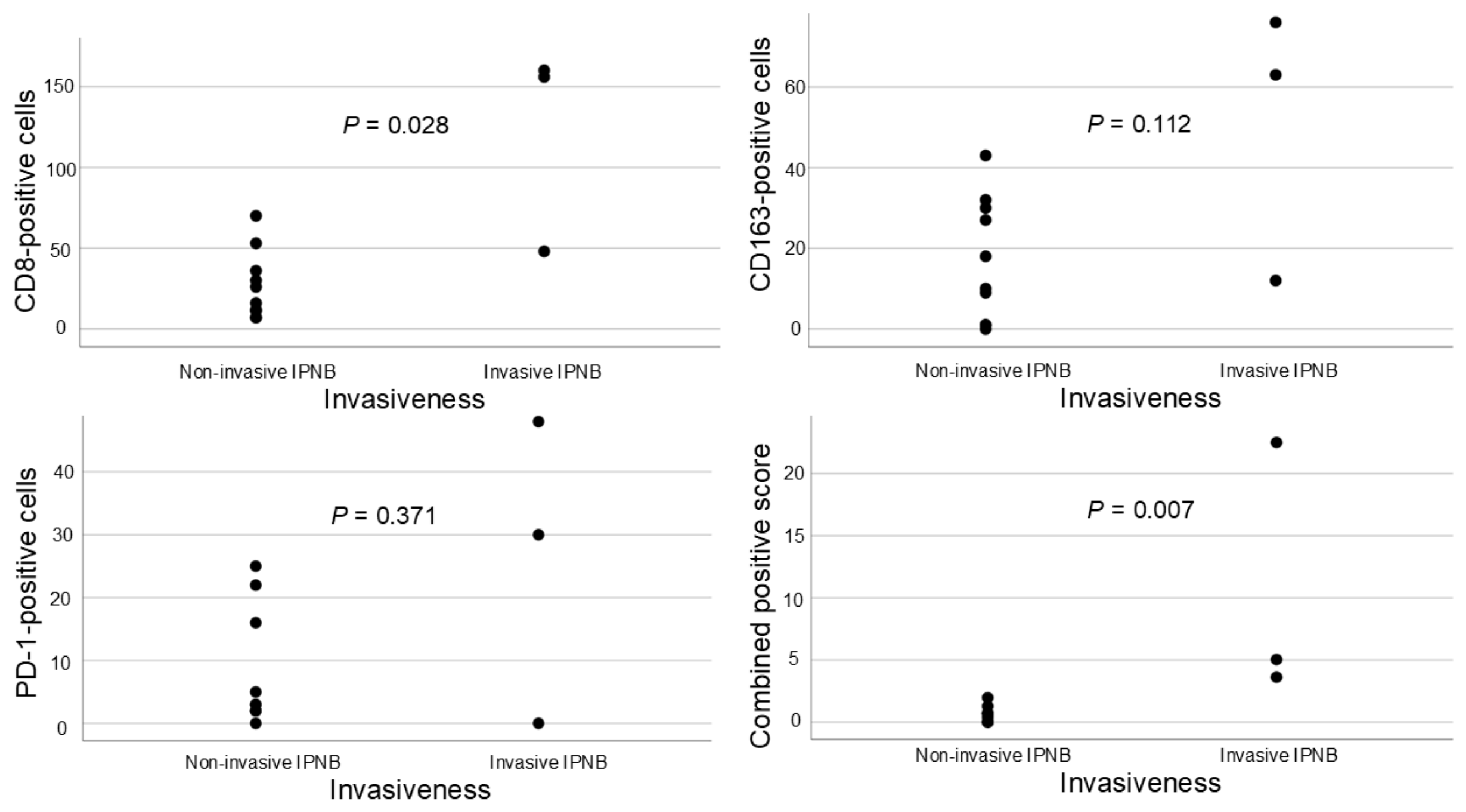
Comparison of the tumor immune microenvironment between invasive and noninvasive IPNBs. Invasive lesions demonstrated significantly higher CD8-positive cell infiltration and PD-L1 CPS than noninvasive lesions.

Invasive lesions demonstrated significantly higher CD8 expression in the TIME (*p* = 0.028) and CPS (*p* = 0.007) compared with noninvasive lesions. However, no significant differences were observed in CD163 or PD-1 expression in the TIME (Figure 4).

### 3.5. Association Between the Tumor Size and the TIME

**Figure 5:**
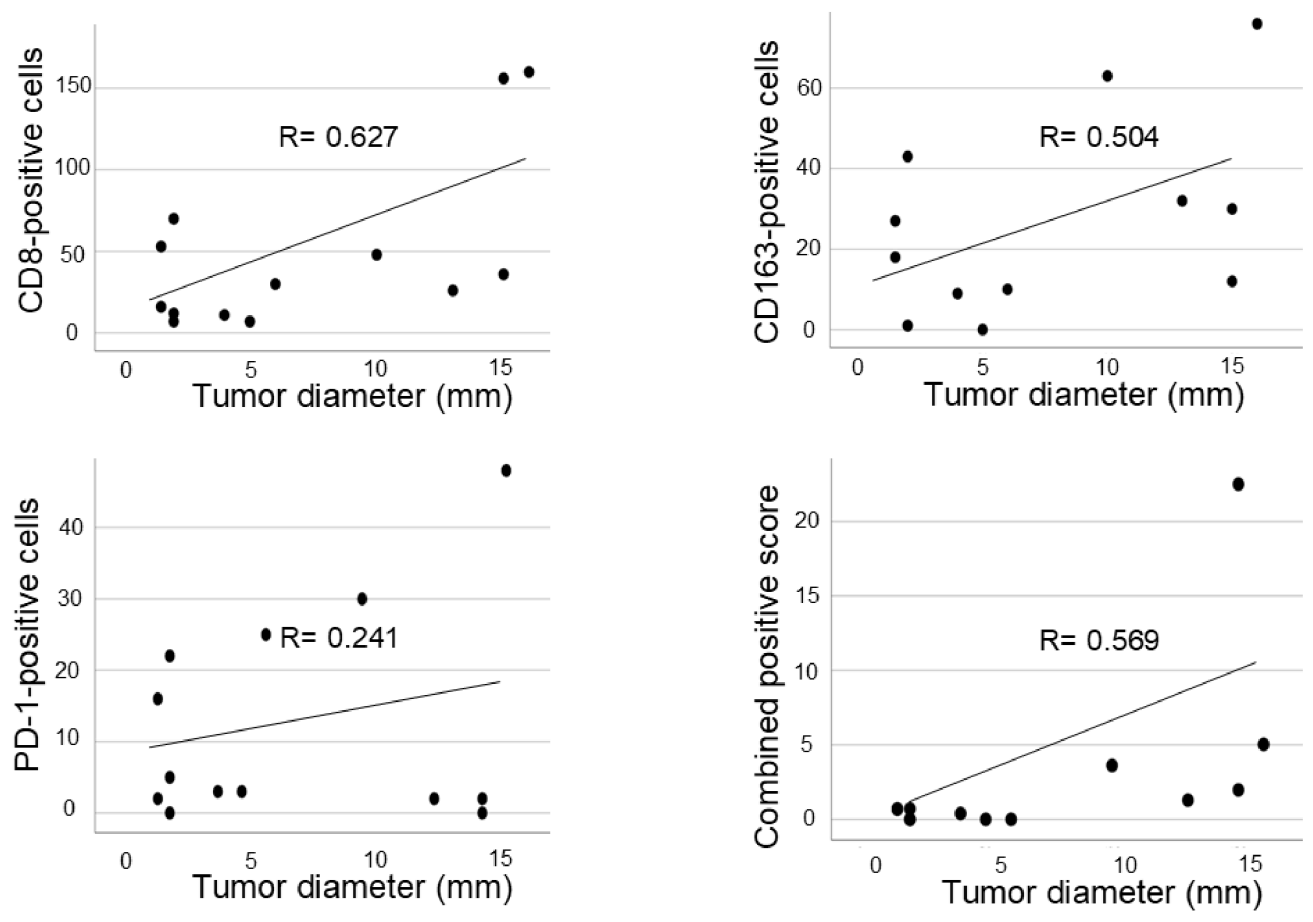
Correlation between tumor diameter and immune-related markers in the tumor immune microenvironment. The tumor diameter was positively correlated with CD8- and CD163-positive cells and PD-L1 CPS. However, no significant correlation was observed with PD-1-positive cells.

**Figure 6:**
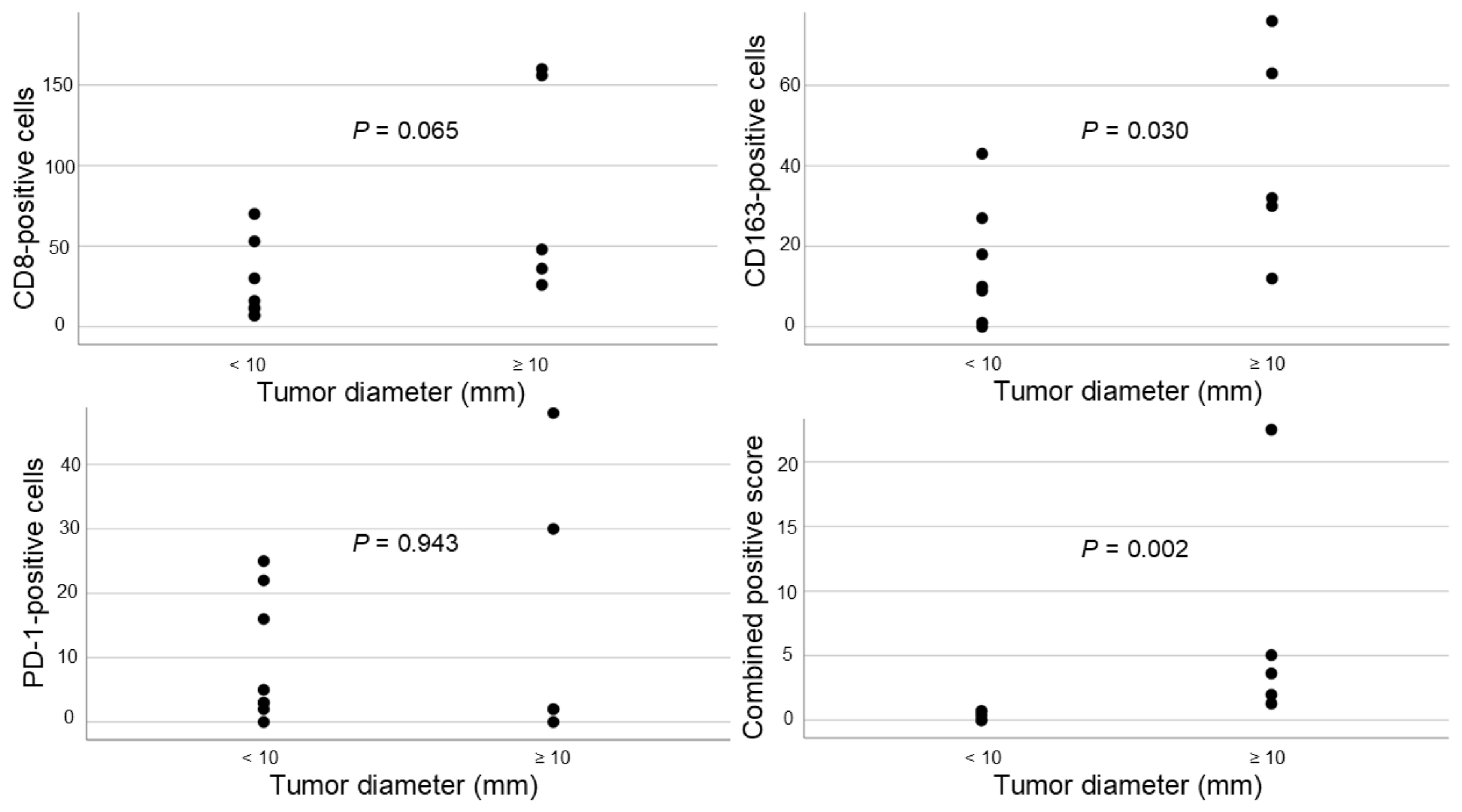
Comparison of immune-related markers according to tumor size of IPNBs (<10 vs. ≥10 mm). Lesions ≥ 10 mm in size demonstrated significantly higher CD163-positive cell infiltration and PD-L1 CPS than those <10 mm in size. CD8-positive cell infiltration tended to be higher in lesions ≥10 mm in size.

The tumor size was positively correlated with CD8- (R = 0.627) and CD163-positive cell count (R = 0.504) and CPS (R = 0.569) (Figure 5). When stratified by tumor size (<10 vs. ≥10 mm), lesions ≥ 10 mm in size showed significantly higher CD163-positive cell count (*p* = 0.030) and CPS (*p* = 0.002) than those <10 mm in size. Moreover, the CD8-positive cell count tended to be higher in IPNBs ≥10 mm in size than those <10 mm in size (*p* = 0.065) (Figure 6). All lesions with CPS ≥ 1 were ≥10 mm in size (*p* < 0.001). No invasive lesions were observed in tumors <10 mm in size (Table 2).

**Figure 7:**
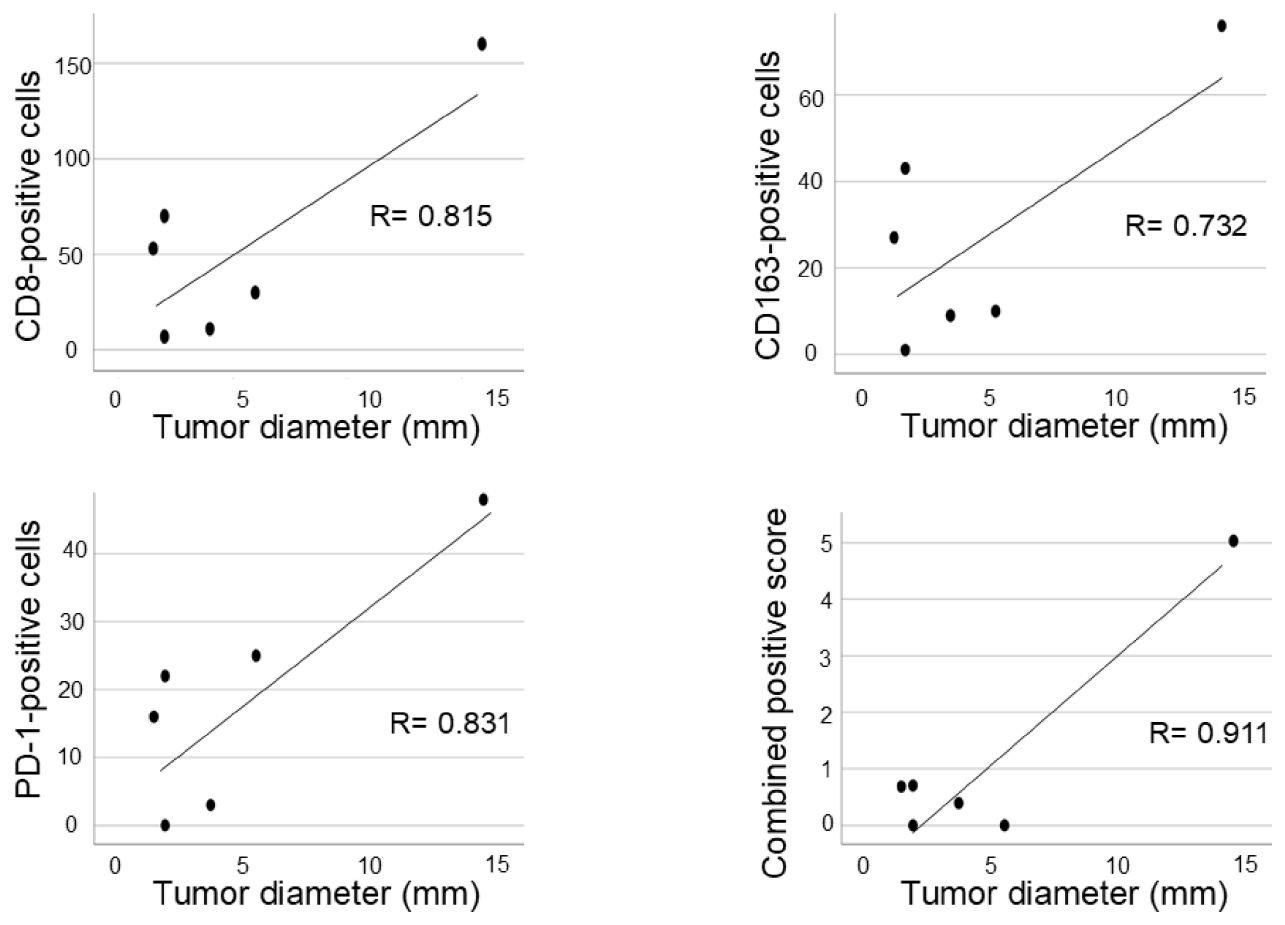
Correlation between tumor diameter and immune-related markers in multiple IPNB lesions from the same patient (patient no. 4). Positive correlations were observed between tumor diameter and CD8-, CD163-, and PD-1-positive cells and PD-L1 CPS, suggesting progressive immune microenvironmental remodeling during tumor enlargement even within the same patient.

Notably, the analysis restricted to multiple lesions within the same patient (patient no. 4) demonstrated positive correlations between tumor size and all immune markers (R = 0.732–0.911) (Figure 7 and Figure S1).

## 4. Discussion

This study comprehensively analyzed the TIME of IPNBs arising in patients with occupational cholangiocarcinoma. The main findings are as follows: (1) tumor size was positively correlated with higher CD163, CD8, and PD-L1 expression in the TIME; (2) lesions ≥ 10 mm in size demonstrated significantly higher CD163 and PD-L1 expression than those <10 mm in size; (3) lesions with CPS ≥1 were ≥10 mm in size; (4) invasive lesions exhibited significantly higher CD163 and PD-L1 expression and were categorized as type 2 IPNBs; and (5) similar size-dependent immune alterations were observed even within lesions from the same patient. Together, these findings suggest that IPNB enlargement is associated with progressive remodeling of the immune microenvironment, particularly involving macrophage infiltration and PD-L1 upregulation. To the best of the authors’ knowledge, this is the first study to demonstrate size-dependent alterations in the immune microenvironment among multiple IPNB lesions within the same patient (under an identical immune circumstance), highlighting intrapatient heterogeneity and suggesting a stepwise immunological evolution during IPNB progression.

One of the most striking observations in the present study is the size-dependent alteration in the TIME. Although previous studies have reported substantial heterogeneity of the immune microenvironment in cholangiocarcinoma, occupational cholangiocarcinoma is characterized by a high TMB and prominent cancer immunoediting across cases [12,14,15]. Despite this background, the present analysis demonstrated that small IPNB lesions (<10 mm) showed neither increased PD-L1 expression nor marked macrophage infiltration. Importantly, similar size-dependent changes in the immune microenvironmental were also observed among multiple lesions within the same patient, suggesting that these findings were not solely explained by interpatient heterogeneity. Based on these findings, immune checkpoint activation in IPNB is not solely determined by the underlying mutational background but rather emerges during tumor progression. Research has shown that genetic alterations associated with malignant transformation, similar to those observed in invasive IPNB, are already present even in micro-IPNB lesions [7,12]. In this context, the present findings raise the possibility that IPNB enlargement reflects progressive immune recognition of tumor-associated molecular alterations by the host immune system, leading to TIME remodeling, including macrophage infiltration and activation of immune escape pathways. The significant correlations between tumor size, CD163-positive macrophage infiltration, and PD-L1 CPS further support this hypothesis. Moreover, all lesions with a PD-L1 CPS ≥1 were ≥10 mm in size. According to previous studies, intrahepatic IPNBs measuring > 12 mm are associated with an increased risk of malignancy [18], and a 10-mm threshold is widely accepted as a predictor of malignancy in gallbladder polyps [19,20,21,22,23,24]. Considering these previous findings together with this threshold’s clinical practicality, we used a 10-mm cutoff to evaluate our cohort. Using this clinically relevant cutoff, lesions ≥10 mm showed a significant association with a PD-L1 CPS ≥1, supporting this threshold’s clinical relevance in evaluating immune microenvironmental changes during IPNB progression.

In the combined IPNB cohort, type 2 IPNBs demonstrated higher immune cell infiltration compared with type 1 IPNBs. Furthermore, all invasive lesions in the occupational IPNB cohort belonged to type 2 IPNBs. These findings support the evidence that type 2 IPNBs may have greater malignant potential [2,3,4]. The present data extended this concept by suggesting that type 2 lesions may also exhibit a more immunologically active and potentially immunosuppressive microenvironment. Recent studies have analyzed differences in the TIME between histological subtypes of intrahepatic cholangiocarcinoma (small- and large-duct types), suggesting substantial heterogeneity within its microenvironment; this implies that appropriate treatment strategies may vary between small- and large-duct-type intrahepatic cholangiocarcinoma [25,26]. Similarly, in premalignant IPNB lesions, there may also be histological subclassification-dependent diversity in the TIME. However, because tumor size, invasive status, and type 2 histology were closely linked in this cohort, further investigation is needed to determine the independent contribution of each factor.

IPNB is recognized as a premalignant lesion of cholangiocarcinoma [1]. Currently, IPNB is generally managed according to the treatment strategies for cholangiocarcinoma [27,28,29,30]. However, uniformly applying highly invasive procedures, including hepatectomy with lymph node dissection or pancreaticoduodenectomy, to all IPNB cases may represent overtreatment. In this context, the present findings regarding TIME alterations observed using a 10-mm cutoff may provide additional insights into the biological progression of IPNB. A recent study demonstrated that intrahepatic IPNBs >12 mm are associated with an increased risk of malignancy [18]. Consistent with this observation, all invasive lesions in the present cohort were ≥10 mm in size. However, the sample size was limited. These findings suggest that the tumor size may be associated with immune microenvironmental changes and progression toward malignancy. Therefore, a 10-mm cutoff may represent a practical threshold for future risk stratification of IPNB, rather than an immediate criterion for clinical decision-making. Although these findings may be particularly relevant in high-risk populations (e.g., patients with occupational exposure), larger cohorts are needed to validate their clinical implications. Furthermore, the observed association between tumor progression and immune microenvironmental changes may provide a rationale for future studies investigating the role of immune-targeted strategies during IPNB progression.

This study has several limitations, including its retrospective design, the relatively small sample size, and the fact that the analysis was confined to patients with occupational cholangiocarcinoma. In addition, tumor size, invasive status, and histological subclassification were closely associated in this cohort, making it difficult to distinguish each factor’s independent contribution to the observed immune microenvironmental changes. Therefore, the present findings should be interpreted with caution and require validation in larger independent cohorts. Despite these limitations, the present findings provide important preliminary evidence suggesting an association between tumor size and immune microenvironmental changes during IPNB progression. Moreover, occupational cholangiocarcinoma provides a unique opportunity to evaluate multiple IPNB lesions even within a limited number of cases. In particular, the ability to analyze the TIME across multiple lesions within the same patient represents a significant strength of this study as it minimizes interpatient variability and allows for a more precise assessment of tumor-progression-associated immune remodeling.

## 5. Conclusions

In IPNB arising from occupational cholangiocarcinoma, tumor enlargement was associated with progressive immune microenvironmental changes, characterized by increased CD8- and CD163-positive immune cell infiltration together with PD-L1 expression. Lesions ≥ 10 mm in size may represent a practical threshold for evaluating immune microenvironmental changes during IPNB progression. However, further studies are needed to validate these findings and clarify the biological and clinical significance of the observed immune microenvironmental changes.

## Figures and Tables

**Table 1:** Background characteristics and pathological findings of 13 IPNBs in 6 patients.

Patient No.	Age	Organic Solvents	Primary Treatment	Main Tumor	Lesions No.	Tumor Diameter (mm)	Subclassification	Phenotype	Invasiveness
1	39	DCP	Surgery	Intrahepatic IPNB with invasive carcinoma	1	15	2	Pancreatobiliary type	Yes
					2	2	1	Gastric type	No
2	39	DCP	Surgery	Intrahepatic IPNB with invasive carcinoma	1	5	1	Pancreatobiliary type	No
					2	1.5	1	Pancreatobiliary type	No
3	31	DCP	Surgery	Mass-forming type intrahepatic cholangiocarcinoma	1	10	2	Oncocytic type	Yes
4	34	DCP	Surgery	Intrahepatic IPNB with invasive carcinoma	1	16	2	Oncocytic type	Yes
					2	6	1	Gastric type	No
					3	4	2	Pancreatobiliary type	No
					4	2	1	Gastric type	No
					5	2	2	Pancreatobiliary type	No
					6	1.5	2	Pancreatobiliary type	No
5	42	DCP, DCM	Chemotherapy	Distal cholangiocarcinoma	1	13	1	Gastric type	No
6	47	DCP, DCM	Surgery	Intrahepatic IPNB with invasive carcinoma	1	15	1	Pancreatobiliary type	No

DCP, 1.2-dichrolopropane; DCM, dichrolomethane; IPNB, intraductal papillary neoplasm of the bile duct.

**Table 2:** The association between tumor diameter and combined positive score.

Variables, *n*		Tumor Diameter (mm)	
		<10	≥10
CPS	<1	8	0
	≥1	0	5

CPS, Combined positive score. *p* = 0.0008.

## Data Availability

All data generated or analyzed during this study are included in this article and its Supplementary Material files. Further enquiries can be directed to the corresponding author.

## Supplementary Materials

The following supporting information can be downloaded at: https://www.mdpi.com/article/10.3390/cancers18162539/s1, Figure S1. Representative immunohistochemical findings of PD-L1 expression in patient no. 4. A small lesion (lesion no. 4-6; diameter, 1.5 mm) demonstrated minimal PD-L1 expression (CPS 0.7), whereas a larger lesion from the same patient (lesion no. 4-1; diameter, 16 mm) demonstrated higher PD-L1 expression (CPS 5.0).

## Abbreviations

The following abbreviations are used in this manuscript:

CPS	Combined positive score
IPNB	Intraductal papillary neoplasm of the bile duct
PD-1	Programmed death protein 1
PD-L1	Programmed death ligand 1
TIME	Tumor immune microenvironment
TMB	Tumor mutational burden
