# Supplementary material for: Immune Microenvironmental Analysis of Intraductal Papillary Neoplasms of the Bile Duct in Occupational Cholangiocarcinoma: Associations with Tumor Size
Source: Cancers (Basel). 2026 Aug 7;18(16):2539. doi: 10.3390/cancers18162539 (PMC13510378; doi:10.3390/cancers18162539)

Supplementary Figure S1

Lesion no. 4-6, Diameter 1.5 mm, PD-L1 CPS 0.7

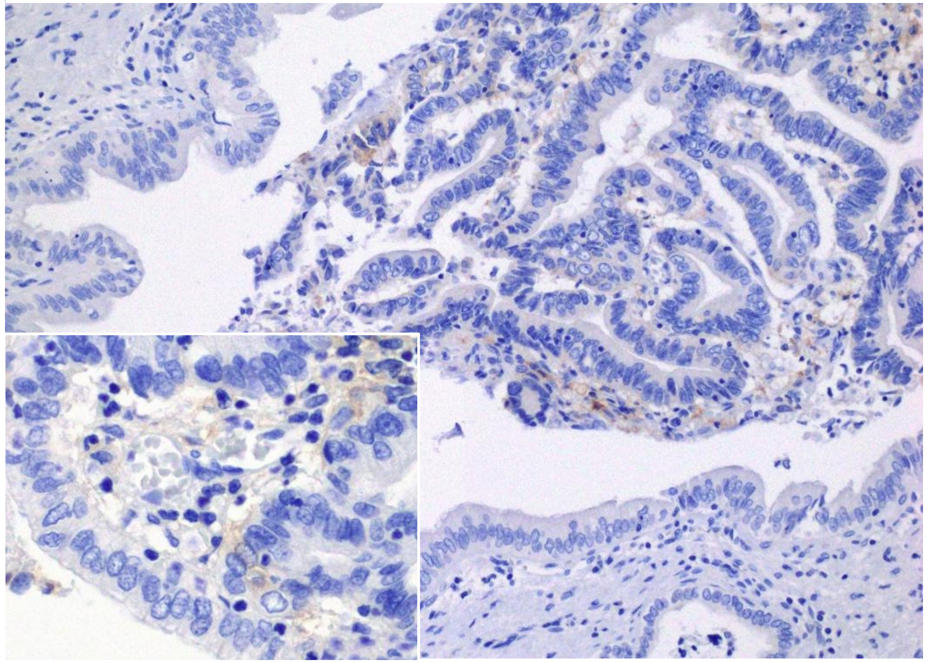

Lesion no. 4-1, Diameter 16 mm, PD-L1 CPS 5.0

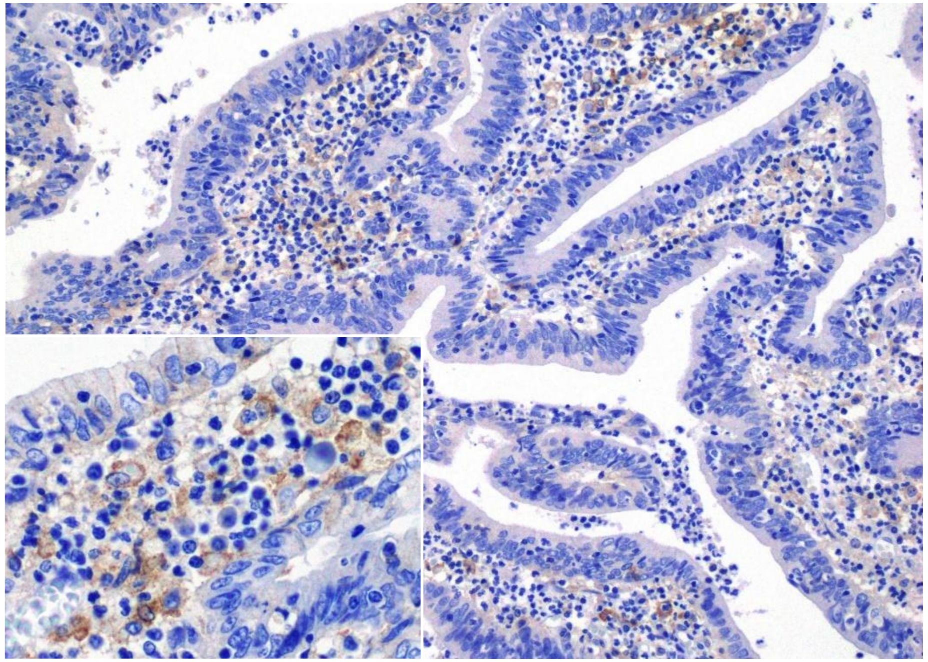

Supplement: Supplementary file 1 [file cancers-18-02539-s001.zip › cancers-4482862-supplementary.pdf]
